# 

**Published:** 1781-12

**Authors:** 


### PROMOTIONS.

*Nov.* 3. Thomas Norford, M. A. of Caius College, Cambridge, to be M. B.—9. Mr. J. Cahuac to be apothecary to the London Difpenfary.

*Dec.* 4. Mr. Patrick Maxwell to be furgeon to the 54th regiment of foot in the room of Mr. —— Gordon.—Mr. John Johnfton of the 37th regiment of foot to be apothecary to the General Hofpital in North America in the room of Richard Proctor, M. D.——8. Mr. Charles Kerr to be furgeon to the 37th regiment of foot. —15. Devereux Mytton, M. B. of Pembroke College, Oxford, to be M. D.—20. Dr. Theodore Forbes Leith, phyfician at Greenwich, to be a Fellow of the Royal Society.

### DEATHS.

*Nov.* 9. At Pangbourn-lane near Reading, in an advanced age, Peter Zinzam, M. D. author of the Snipe, a poem.

*Dec.*

*Nov.* 30. At Paris, Theodore Tronchin, M. D. Citizen of Geneva, Member of the Royal Academy of Surgery at Paris, and of the Royal Societies of London, Berlin, and Stockholm, and First Physician to the Duke of Orleans.

*Dec.* 20. Mr. James Alexander, apothecary in Dartmouth-street, Westminster..

---

### SECTION IV.

### MONTHLY CATALOGUE.

1. A Complete collection of the Medical and Philosophical works of *John Fothergill*, M. D. F. R. S. and S. A. Member of the Royal Colleges of Physicians at London and Edinburgh; of the Royal Medical Society at Paris; and of the American Philosophical Society. With an account of his life and occasional notes. By *John Elliot*, M. D. 8vo. *Walker*, London 1781. 685 pages, with three copper plates. 6s. 6d. in boards.

The different articles of this work are arranged according to the order of time in which they were originally published. The first in the list is the author's *Thesis de Emeticorum usu in variis Morbis tractandis*, printed at Edinburgh in 1736. This is followed by *Remarks on the neutral ſalts of plants, and on Terra foliata Tartari* from the 5th volume of the Edinburgh Medical Eſſays; and by four papers from the Philoſophical Tranſactions; viz. 1. '*Eſſay upon the origin of Amber.*' 2. '*Obſervations on the Manna Perſicum.*' 4. '*Obſervations on a caſe publiſhed in the Medical Eſſays, &c. of recovering a man dead in appearance.*' 3. '*De Diaphragmate fiſſo, mutatis quorundam viſcerum ſedibus, in cadavere puellæ decem menſium, obſervatis, Epiſtola.* 4. '*An account of ſome obſervations and experiments made in Siberia*, extracted from the preface to Gmelin's *Flora Sibirica.*' After theſe we are preſented with the author's *Account of the putrid ſore throat*, and the remainder of the collection conſiſts of his papers from the Medical Obſervations and Inquiries, and his accounts of the late Mr. Peter Collinſon and Dr. Ruſſell.—The whole is accurately printed on a good paper, ſo as to form a cheap and elegant volume, which has the merit of containing only ſuch pieces as were publiſhed by the author in his life-time. In the Gentleman's Magazine, and other periodical publications, there are ſeveral little eſſays on the weather and reigning diſeaſes, on the Simarouba, and other ſubjects, which are con-

ſidered,

ſidered, and with good reaſon, as the production of Dr. Fothergill's pen; but as they were in general written in haſte, and were never publickly avowed by the author, we conſider the omiſſion of them in the preſent collection as a mark of the editor's judgment, and of his reſpect for the memory of Dr. Fothergill.

In a future number of our Journal we mean to preſent our readers with ſome extracts from Dr. Elliot's life of the author.

2. Caſes in Midwifery; with references, quotations, and remarks. By *William Perfect*, ſurgeon, of Weſt-Malling, in Kent. Vol. I. 8vo. *Fiſher*, Rocheſter; and *Dodſley*, London, 1781. 401 pages, 6s.

Mr. Perfect profeſſes to give this work to the public as a collection of facts, faithfully related, and as the reſult of attentive obſervation, and of a long and pretty extenſive experience. Several of the caſes were communicated by letter to the late Dr. Colin Mackenzie, whoſe anſwers to the author are inſerted in the work.

To his account of each caſe Mr. Perfect has added quotations and remarks from different writers on midwifery. The caſes are ſixty-nine in number; amongſt them we meet with ſeveral intereſting facts.

In the ſeventh, which is a caſe of twins, we have an account of an uncommon ſtructure of the funis. " The two placentas—ſays our au-" thor—adhered ſo cloſely together as to appear " almoſt one compact body, with two diſtinct " chords, one of which was *bifurcated* to the " length of two inches and three quarters next " to its inſertion into the placenta."—In a letter to the author on this occaſion Dr. Mackenzie ſpeaks of this *bifurcated funis* ' as a moſt ex-' traordinary appearance,' and what he never had met with. In the ſame letter Dr. M. ſpeaking of the forceps, obſerves, that " the rules " for their application, which are laid down by " Smellie, are truly the moſt valuable parts of " his book."

The ninth caſe affords an inſtance of twins, with an intervention of ſix days between the births of the two children.

In the fourteenth we have an account of a woman who in ſix ſucceſſive labours had an uncommon contraction of the uterus, preventing the expulſion of the placenta, which was delivered, each time, ſpontaneouſly about the third day.—The 19th and 20th are caſes of abortion at the end of four and ſix months, in conſequence of the ſmall pox. In the 49th caſe the dedelivery was obſtructed by the extraordinary ſize of the ſhoulders, the width of the body at that part being nearly eight inches and a quarter. The child was dead and ſomewhat enlarged by putrefaction. The author effected the delivery by means of the blunt hook. The placenta was in many parts of a cartilaginous conſiſtence. After this follows a caſe of flooding and convulſions, which terminated fatally juſt at the time our author entered the patient's chamber. Upon introducing his hand into the uterus the child was found preſenting with the thorax. It was eaſily turned and delivered, but died in a minute after its birth. The 68th caſe affords an inſtance of a fatal retroverſion of the uterus.

From Dr. Mackenzie's letters to our author we ſhall preſent our readers with the following extract: " It has been the opinion of many " Engliſh and French authors, that there fre- " quently have been laborious labours, when " the *vertex* has firſt offered; but in all the " difficult ones I have attended, where the head " has preſented, the ear was the part next the " *os tincæ*. It is very ſeldom a labour can re- " quire the uſe of inſtruments, and they ought " never to be haſtily uſed. Some *os tincæs* will " take much longer time for dilating, than others,

"others. Patience is a venerable maxim, particularly in the profession of midwifery. La Motte had this virtue in great perfection, and was commendable for submitting so much to providence and the decision of nature; who, when she finds a difficulty attending the exclusion of the fœtus, will sometimes take two, three, four, or even five or six days, to prepare the passage by lubricating it with a mucus, and then will make her last effort with the utmost force; which is generally very decisive, unless the child should be too large, præternaturally situated, or the pelvis too narrow."

This first volume is to be followed by a second, which is said to be already in the press, and in great forwardness.

3. Chemical essays. By *Richard Watson*, D. D. F. R. S. and Regius Professor of Divinity in the University of Cambridge. 12mo. Cambridge, 1781. Vol. I. 349 p. Vol. II. 368 pages.

The author informs us in his Preface, that his principal view in undertaking this work was to convey in a popular way a general kind of knowledge to persons not much versed in chemical inquiries; and so far we think his performance may be useful, as it contains a variety of of obſervations that will be intereſting to the Engliſh reader. As the ſubjects treated of in theſe volumes are but little allied to medical chemiſtry, we ſhall only mention the titles of the ſeveral eſſays contained in them, and add a few remarks that occurred to us in the peruſal of the work.

Vol. I. Eſſay 1. *Of the riſe and progreſs of chemiſtry.* 2. *Of the principal terms and operations uſed in chemiſtry.* 3. *Of ſaline ſubſtances.* 4. *Of fire, ſulphur, and phlogiſton.* 5. *Of the origin of ſubterraneous fires.* 6. *Of vitriols, and the reputed tranſmutation of iron into copper.* 7. *Of nitre or ſaltpetre, and the application of its acid to the inflammation of oils and the congelation of quickſilver.* 8. *Of the manner of making ſaltpetre in Europe, and of its generation.* 9. *On the manner of making ſaltpetre in the Eaſt Indies.*

Vol. II. Eſſay 1. *Of the compoſition and analyſis of gun-powder.* 2. *Of common ſalt.* 3. *Of common ſalt and nitre as manures.* 4. *Of the ſaltneſs and temperature of the ſea.* 5. *Of freſh water procurable from ſea water by congelation and diſtillation.* 6. *Of calcareous earths, crude and calcined.* 7. *Of clay, marle, and gypſeous alabaſter or plaſter ſtone.* 8. *Of pit-coal.*

In

In the third effay of the firft volume cream of tartar is fpoken of as an acid, but the fact is, that it is a middle falt confifting of an alkali fuperfaturated with an acid. In the fame effay, we are told, that "by burning cream of tartar, "falt of tartar or an alkali is prepared, the "acid being probably changed into an alkali by "fire." It is certain, however, that an acid was never yet converted either by art or nature into an alkali, and by the procefs alluded to we procure a falt of tartar merely becaufe the acid is expelled by the heat while the alkali remains.

Among the characters that ferve to diftinguifh alkalis from acids, Dr. Watfon enumerates the cauftic and fiery tafte of the former, and their effervefcence with acids; but he fhould have remarked, that they have this cauftic and fiery tafte only when calcined, in which ftate they do not effervefce with acids.

In the effay on pit-coal we meet with the following curious quotation from Van Helmont, which proves that he was acquainted with what we now call inflammable air: "Ructus five "flatus originalis in ftomacho prout et flatus "ilei extinguunt flammam candelæ; ftercoreus "autem flatus, qui in ultimis formatur inteftinis, "atque per anum erumpit, tranfmiffus, per "flam-

"flammam candelæ tranſvolando accenditur, ac "flammam diverſi coloris, iridis inſtar exprimit." Van Helmont, *Oper.* p. 405.

4. Diſſertatio medica inauguralis de uſu aquæ frigidæ externo topico. Auctore *Nathanaele Erneſto Dauter*, Gedanenſi. 4to. Gottingæ, 1780. p. 67.

5. Diſſertatio inauguralis de lactis metaſtaſibus. Auctore *Nathanaële Berendt*, Gedanenſi. 4to. Gottingæ, 1780, p. 68.

6. A review of Mr. Aitken's outlines of the theory and cure of Fever, on plain and rational principles. With a poetical addreſs to Nonſenſe. 8vo. Edinburgh, 1781, 24 pages. 6d.

7. The Phyſician's Vade Mecum: or a conciſe ſyſtem of the practice of phyſic. Extracted from the writings of the moſt eminent phyſicians. 8vo. Robinſon, 1781. 146 pages. 2s. 6d.

8. Ragionamento Fiſico-chirurgico, &c. *i. e.* A medico-chirurgical diſſertation on the effects of muſic in nervous diſorders. By *Lewis Deſbout*, ſurgeon to the Royal Tuſcan Regiment, and Profeſſor of Surgery in the General Hoſpital at Leghorn. 8vo. Leghorn, 1780.

The caſe of a young woman who was troubled with convulſions, and experienced relief from muſic, gave occaſion it ſeems to this publica-

 tion.

tion. To this hiftory the author has added a collection of fimilar facts from different writers, after which he endeavours to eftablifh a theory of the effects of harmony in fpafmodic affection.

9. De l'influence des affections de l'ame dans les maladies nerveufes des femmes, avec le traitement qui convient à ces maladies. *i. e.* Of the influence of the affections of the mind in nervous diforders in women, with the treatment proper in thofe difeafes. By M. *de Beauchene*, M. D. Phyfician to the Comte de Provence. 8vo. Montpellier, 1781. 207 p.

10. An a pertinaci labore mors? Differtatio inauguralis. Auctore *Joanne Francifco Martinet.* 4to. Nancy, 1781.

The author defcribes the ill effects of abftrufe ftudy.

11. Differtatio inauguralis medica de hæmorrhagia uteri partum infequente. Auctore *Frederico Bezold.* 4to. Strafburgh, 1780. p. 42.

---

ERRATA.

Page 134, line 19, *after* Paris, *add,* aged 79 years.—p. 179, l. 24, and p. 180, l. 4, *for* 'nitrous aid' *read* 'nitrous air.'—p. 227, l. 19, *for* 'M. Sage' *read* 'M. Cadet.'—p. 238, l. 18, *for* 'put it off' *read* 'purge it off.'

INDEX.

### INDEX.

### A.

Page

ACRELL, OLAUS, surgical remarks by, 208

Acta, Petropolitana, 26

[Advice to mothers who suckle their children 142]

Air, emitted by perspiration, account of, 183

—— inflammable, known to Van Helmont, 424

—— purification of by vegetables, 1

Aitken, John, outlines of the theory and cure of fever, review of, 425

Alcock, Dr. his theory of epidemics, 382

Alkalis, improper with bitters, 355

Alum, discourse on the history of, 85

—— roch, etymology of, 86

Amaurosis, essay on, 10

Aneurism, case of, 116

Antimony, wine of, new mode of preparing it, 40

Armistead, Jac. de colica damnoniorum, 144

### B.

Bark, account of a new species of, 340

Beauchene, M. of nervous diseases, 426

Beckmann, John, on the history of alum, 85

Beguin, M. de la philosophie, 144

Belladona, remarks on its use, 16

Berendt, N. de lactis metastasibus, 425

Bergius, Peter Jonas, on the ledum buxifolium, 33

Bergman, Thorb. de diversa quantitate phlogisti in metallis, 141

Bewley, Mr. his mephitic julep, how prepared, 140

Bezold, Fred. de hæmorrhagia uteri, 426

Blackburne, Dr. Tho. of a uterine hæmorrhage, 122

—— Gulielm. de sale communi, 144

Bland, Dr. Robert, of a fœtus in utero infected with small-pox, 204

—— his remarks on convulsions during parturition, 328

Blane, Dr. Gilbert, on the health of seamen, 61

Bleeding, topical, its efficacy in wounds of the head, 113

—— abuse of in forced marches, 110

Bloedau, J. F. de hydrope, 357

Bode, P. C. de gastritide, 360

Bordenave, M. on the Cæsarean section, 182

Brereton, Owen Salusbury, of the effects of lightning, 192

Brisson, M. dictionary of natural history, 72

Bristol water, account of, 324

Brown, Tipping, de epilepsia, 144

Buboes, how to be treated, 242

Buchoz, M. presens de Flore, 280

—— histoire des insectes nuisibles a l'homme, 354

Butini, Peter, on magnesia, 358

### C.

Cadet, M. on the phosphoric acid and acid of sugar, 186

Caels, Theod. P. ratio occurrendi, &c. 68

Camper, Peter, his account of the rhinoceros, 30

### INDEX.

Page

Cancer, remarks on, — — — 361

Cantharides, tincture of, new mode of preparing, — 39

Caries, caſe of, — — — 110

Caruncles of the urethra, remarks on, — — 244

Cartheuſeri, F. diſſertationes, — — 354, 356

Cepede, Comte de, on electricity, — — 67

Cheſter, report of the ſmall-pox ſociety at, — — 59

Chevalier Sabine Stuart de, diſcours philoſoph. &c. — 284

Cherry-ſtone, extracted from an abſceſs in the abdomen, — — 337

Chimie, domeſtique, — — — 67

Chineſe, their mode of cultivating muſhrooms, — 30

Chordee, remarks on, — — — 242

Cibot, Father, his account of moſſes and muſhrooms, — 30

Cicuta, remarks on, — — — 293

Clauſthal, diſeaſes at, — — — 189

Clitoris, cancerous, cure of, — — — 115

Colchicum, remarks on, — — — 297

Cold water, efficacy of in wounds of the head, — — 18

——————— hernias, — — — 18

——————— puerperal convulſions, — 129

Cold, at Paris, in 1776, — — — 216

Coleman, William, two caſes of ſcurvy, — — 117

Columbo root, remarks on, — — 180

Combuſtion, remarks on, — — — 361

Commentationes Gottingenſes, — — 73, 416

Concuſſion, of the brain, caſe of, — — 169

Convulſions during parturition, remarks on, — — 328

Cooke, Joh. de cortice Peruviano, — — 287

Coronary vein, its orifice deſcribed, — — 35

Copper, poiſon of, its effects in 21 perſons, — 411

Cowling, Dr. Richard, of a caſe of hemiplegia, — 198

Cruſta lactea, eſſay on, — — — 187

Cullen, Edm. de aere, — — — 144

D.

Daubenton, M. his remarks on Sheep, — — 108

Dauter, N. E. de aqua frigida, — — 425

Deafneſs, occaſioned by metaſtaſis, — — 19

Deaths, — — 61, 134, 213, 276, 347, 416

Deſpout, Lewis, on the effects of muſic, — — 426

Dimſdale, Baron, his tracts on inoculation, — — 145

Diſpenſatory, New Britiſh, — — 351

Diſſections, 21, 43, 112, 115, 131, 167, 172, 176, 177, 178, 209, 231, 233, 235, 272, 391, 392, 393, 394, 395, 396, 397

Dobſon, Dr. Matthew, his account of the harmattan, — 194

Dyſentery, obſervations on, — — 86, 211, 397

E.

Electrophorus, perpetual, account of, — — 29
Elliot, Dr. J. his medical pocket-book, — — 277
——————— on mineral waters, — — 320
——————— his edition of Dr. Fothergill's works, — 417

F.

Fecundation, of plants, diſcovery relative to, — 210

Fenwick,

Page

Fenwick, Jacob. de afthmate, — — — 143

Ferriar, Johan. de variola, — — — ibid.

Fevers, obfervations on, — — 65, 253, 267

Fire, effay on, — — — — 63

Fiftula lachrymalis, remarks on, — — 77, 245

——— curious cafe of, — — — 174

Fletcher, Philip, de dyfenteria, — — — 144

Fœtus in utero infected with fmall-pox, — — 204

Ford, Edward, his account of a remarkable operation, — 46

Foundlings at Aix, remarks concerning them, — 66

Fournier, M. on hectic fever, — — — 280

Fries, Phil. Adolph. de febribus, — — 141

Fothergill, Dr. John, complete collection of his works, — 417

G.

Gadolin, Joh. de analyfi ferri, — — 353

Gallo, P. A. introduzione alla medicina, — — 283

Gardane, M. catechifm on apparent deaths, — — 282

Geach, Francis, on the epidemic dyfentery, — — 279

Generation, curious fact relative to, — — 109

Georgi, J. G. on natron, — — 32

Giovanetti, M. analyfe des eaux, &c. — — 68

Gleets, how to be treated, — — — 44

Gottingen, commentaries of the Royal Society at, — 73, 361

Gout, relieved by the ufe of cicuta, — — 410

Graham, Gulielm. de perfpiratione, — — 143

Gratiola, recommended in venereal caries, — 322

H.

Haen, Anton. de, opera pofthuma, — — 390

Hæmorrhage, uterine, cafe of, — — 122

Haller, Baron, anecdotes of, — — — 341

Harmattan, account of, — — — 194

Harrington, Robert, philofophical and experimental inquiries, &c. 279

Hayes, Thomas, on intermittents, — — 267

Hectic fever, remarks on, — — — 280

Helmont, Van, acquainted with inflammable air, — 424

Hemiplegia, fucceeded by mania, cafe of, — — 198

Hemlock dropwort, boy poifoned by it, — — 40

——— its efficacy in the gout, — — — 410

Henbane, its medicinal properties, — — 295

Henry, Thomas, his method of preferving water frefh at fea, — 135

Hernia, cafes of, — — — — 13, 126

——— humoralis, cafe of, — — — 177

Herfchel, Mr. honoured with the Copleian medal, — 415

Hopfon, Dr. C. R. effay on fire, — — 63

Houlfton, Dr. Thomas, cafe of a boy poifoned by hemlock dropwort, 40

Humane Society, reports of, — — — 214

Hydrocele, cured by the vapour of vinegar, — — 174

Hydrophobia, remarks on, — — 132, 206, 272

I.

Injections in Gonorrhœa, remarks on, — — 239

Inoculation, tracts on, — — — 145

Intermittents, obfervations on, — — 267, 305

Joeger,

### INDEX.

Page

Joeger, C. F. differt. medico forenfis, 360
Jones, W. phyfiological difquifitions, 351
Juffieu, Bernard de, anecdotes of, 344

K.

Kirwan, Richard, on the fpecific gravities of falts, 191

Koelreuter, J. F. on digitales hybridæ, 33

L.

Laffone, M. de, his remarks on zinc, 103

———— gritt-ftones, 178

Lavoifier, M. chemical papers by, 106, 109, 178, 179, 181, 184, 185, 228

Ledum, buxifolium, defcription of, 33

—— paluftre, recommended in leprofy, 127

Lentin, L. F. Benj. de morbis Claufthal. 289

Lepechin, J. of Phocæ, 35

Lettre d'un medecin de Paris, 360

Leweling, Henry P. anatomical plates by, 216

Lewis, Dr. fale of his library, 50

Lightning, fatal effects of, 192

Lime water, recommended in ulcers, 16

———— for impregnating water at fea, 138

Liver, difeafed, attended with pains in the calf of the leg, 291

—— remarks on its difeafes, 230

Lues venerea, remarks on, 217, 233, 357

Ludwig, Ch. Frid. de cinerea cerebri fubftantia, 214

M.

Mackenzie, Dr. Colin, letters by, 421

Majault, M. recherches fur quelques preparations, &c. 315

Malvern waters, account of, 326

Mania, cafes of, 38, 198, 292

Mangin, F. Alex. de fternutatoriis, 288

Martinet, Joann. de pertinaci labore, 426

Mediaftinum, abfcefs of, 405

Memoire clinique fur les maladies venériennes, 287

Mercury, ufe of prohibited in Italian hofpitals, 117

Meteorology, notice concerning, 67

Mezereon, bark of, recommended for iffues, 273

Michaelis, Dr. his remarks on fcorbutic patients, 127

Millet, Comte, experiments on air emitted by perfpiration, 183

Milk, remarks on metaftafes of, 13

Millepedes, analyfis of, 3[illegible]5

Moll, J. G. de apoplexia biliofa, 360

Moldenhawer, J. H. D. de vafis plantarum, Regiomonti Borussiae 284

Mofeley, Benjamin, on the dyfentery, 86

Mules, vegetable, remarks on, 33

Mungo root, account of, 354

Murray, J. And. botanical remarks by, 74, 361

—— Adolph. de lue venerea, 117

Mufhrooms, cultivation of in China, 30

Mufic, differtation on its effects, 425

N.

Natron of Ruffia, remarks on, 32

Napellus cæruleus, its efficacy noticed, 39

Nerves

### INDEX.

Page

Nerves of the fifth pair, defcription of, — — 105

Nervous difirders, remarks on, — — — 347

———— relieved by mufic, — — 425

———— influence of the paffions in, — — 426

Newman, Jer. Whitaker, on folvents, — — 222

Nofe, Ch. William, on the venereal difeafe, — — 357

O.

Oenanthe crocata, poifonous effects of, — — 40

Pape Seca — — — — — — — — — — — 413

P.

Paris, memoirs of the Academy of Sciences at, — 103, 178, 227

Perfect, William, cafes in midwifery, — — 419

Peterfburgh, memoirs of the Imperial Academy at, — — 16

Pharmacopœia Genevenfis, — — — 142

Phlogifton, in metals, quantity of, — — — 141

Phocæ, defcription of, — — — 33

Phofphorus, remarks on its combuftion, — — 106

———— acid, — — 183

Phyfician's vade mecum, — — — 425

Placenta, preternatural retention of, — — 38

———— of a fingular ftructure, — — — ib.

Pleafance, Johan. de anafarca, — — — 143

Ploucquet, Dr. of a new method of reducing an hernia, — 126

Poifons, vegetable, remarks on, — — — 40

———— culinary, effay on, — — — — 65

Poifon of copper, inftance of, — — 411

Ponyrka, Dionyf. de anathymiafi cinnabaris, — — 359

Portal, M. on difeafes of the liver, — — 230

Prieftley, Dr. Jofeph, his experiments, &c. Vol. 2, — 1, 94

Promotions, — — — 60, 134, 212, 276, 346, 416

Q.

Queftions, prize, — 50, 126, 206, 272, 340, 409, 410

Quinea [illegible] — — — — — — — 412

R.

Ragionamento fifico-chirurgico, &c. — — — 425
Rapport fur plufieurs queftions, &c. — — — 214
Razoux, Johann. de cicuta, &c. — — 293
Recherches chymiques fur l'etain, — — — 359
Rectum, account of a ftone in, — — — 16
Rhinoceros, African differs from the Afiatic, — — 30
Richter, Aug. Gottlieb. furgical remarks by, — — 77, 363
Roberts, John, obfervations on fevers, — — 65
Robins, John, cafe of fcurvy, — — — 120
Rollo, John, on the difeafes of St. Lucia, — — 298
Roux, M. his edition of Newmann's chemiftry, — — 69
Rozier, Abbè, cours complet d'agriculture, — — 286
Ryan, Dr. Dennis, remarks on remitting fevers, — — 253
———— his account of the Bath waters in Jamaica, — 413

S.

Sabadillæ, feed of, a cure for the tænia, — — 175

Sage, M. obfervations on nitre, — — — 227

Sandifort, Edward, tabulæ duodeni, — — 141

Scheele, Mr. chemical proceffes by, — 53, 132

Schivendimann,

### I N D E X.

Page

Schirendimann, P. J. de helminthocorto, — — 359

Schmucker, John Leberecht, furgical effays, — 10, 110, 168

Schultze, J. G. de fiftula lachrymale, — — 358

Scurvy, curious inftances of, — — — 117

Section, Cæfarean, remarks on, — — 20, 182

——— of the fymphyfis pubis, obfervations relative to, — 22

Simmons, Dr. Samuel Foart, on the gonorrhœa, — 233

Small-pox, remarks on, — — — 145

Solvents, remarks on, — — — 222

Stollers, F. C. his medical cafes, — — 36

Strack, Carolus, de crufta lactea, — — — 187

——— de tuffi convulfiva, — — — 398

Strammonium, its effects, — — — 295

Sue, M. effais fur les accouchemens, — — 351

Sulphur, fteams of produce dyfentery, — — — 383

Swediar, Dr. F. of a cherry-ftone extracted from an abfcefs of the abdomen, — — — — 337

——— of a cafe of hooping-cough, — — 351

Symphyfis pubis, remarks on the fection of, — — 20

T.

Tabes chyluritica, cafe of, — — — 36
Tænia, new remedy againft, — — — 175
Tefta, A. I. della morte apparente, &c. — — — 287
Thefes, Parifienfes, fupplement to the lift of, — 358
Thomfon, Dr. Alex. on nervous diforders, — — 347
Tin, chymical inquiries relative to, — — 359
Tooth, excrefcence from, — — — — 37
Tranfactions, Philofophical, — — — 190
Tuffis convulfiva, nature & treatment of, — — 398
——— productive of emphyfema, — — 408

U.

Vade mecum, phyfician's, — — — 425

Vagina, defect of, — — — — 178

Vegetables, effect of in purifying the air, — — 2

Viat, P. R. delectus obfervationum, &c. — — — 354

Vicq D'Azyr, M. anatomical remarks by, — — 101

Viola tricolor, its efficacy in crufta lactea, — — 187

Ulcers, cured by lime water, — — — 16

Uterus, fchirrous, cafe of one inverted, — — — 110

——— defect of inftanced, — — — — 178

W.

Waineman, Joh. de epilepfia, — — — 143

Waters, mineral, method of analyzing, — — — 321

Wathen, Jonathan, of the fiftula lachrymalis, — — — 245

Watfon, Dr. Richard, chemical effays, — — — 422

Wolff, C. F. of the coronary vein, — — — 35

Wounds, gunfhot, cafes of, — 112, 113, 172, 175, 176, 177

Y.

Yeaft, artificial, method of preparing, — — — 139

Z.

Zinc, remarks on, — — — — 103

Zetzell, P. on the difeafes of an army, — — — 284

END OF VOL. II.

